# Solid pseudopapillary neoplasm of the head of the pancreas: A case report

**DOI:** 10.1016/j.amsu.2021.102708

**Published:** 2021-08-10

**Authors:** Driss Erguibi, Yassine El Berni, Aziz Moufakkir, Rachid Boufettal, Rifki jai saad, Farid Chehab

**Affiliations:** a Faculty of Medicine and Pharmacy, Hassan II University of Casablanca, Casablanca, Morocco; b Department of Digestive Cancer Surgery and Liver Transplantation, CHU Ibn Rochd, Casablanca, Morocco

**Keywords:** Solid pseudopapillary tumor, Pancreatic tumor, Whipple procedure, Case report

## Abstract

**Introduction:**

and importance: Solid pseudopapillary tumor of the pancreas (SPN) or Frantz's tumor is a rare tumor of low malignant potential common in young women. The aim of this paper is to present and discuss a case of a solid pseudopapillary tumor of the pancreas occurring in a 19-year-old female.

**Case presentation:**

A 19-year-old girl presented to our department with epigastric pain for two months, she had no clinical findings on physical examination. Abdominal Computed tomography scan (CT scan) showed the presence of a well-defined tumor arising from the pancreatic head measuring 9,1 × 8.1 × 8.5 cm, heterogeneous and with solid and necrosis components. The patient was subjected to surgery and histopathological examination confirmed the diagnosis of a pseudopapillary tumor of the pancreas.

**Clinical discussion:**

This is an interesting case report of a rare tumor, in so far as without any adjuvant chemotherapy Prognosis of the tumor is better than other pancreatic tumors. surgical resection seems to be the best strategy in the management of SPT.

**Conclusion:**

Close follow up is necessary for early detection of the recurrence and metastasis.

## Highlights

• Solid pseudopapillary tumor of the pancreas is a rare tumor of low malignant potential.
• The prognosis is favorable even in the presence of distant metastasis.
• Surgical resection is the treatment of choice.
• Close follow up is necessary to early detection of the recurrence and metastasis.

## 1 Introduction

Solid pseudopapillary neoplasm (SPN) is a rare epithelial neoplasm of unknown origin, [1,2]. It differs from other pancreatic neoplasms by a clear preponderance in females and a low rate of malignancy; they are typically seen in females in their 20s–30s, but can also be seen in the pediatric population [4]. The neoplasm is also referred to as a Frantz tumor, after its discoverer Dr. Virginia Frantz in 1959 [5]. In 1996, the World Health Organization (WHO) renamed this tumor solid pseudopapillary tumor for the international histologic classification of tumors of the exocrine pancreas [6]. The treatment of choice is complete surgical resection, and it depends on the location of the tumor. The patients with SPN have an excellent prognosis after surgical excision. This case report aims to demonstrate the clinicopathological findings encountered and the management of a patient diagnosed with SPN. This work has been reported in line with the SCARE 2020 criteria [3].

## 2 Case presentation

A 19-years-old female, a university student, and resident in central Morocco were referred to our department due to epigastric pain for 2 months. The pain was progressive and persistent, not radiating without relief factors. There was no history of vomiting, fever, jaundice, or weight loss. The patient had no medical or surgical history and no history of alcohol or drug abuse. The Patient had no significant family history. On general examination vitals were stable, there was no tenderness or palpable mass on examination of the abdomen.

**Fig. 1:**
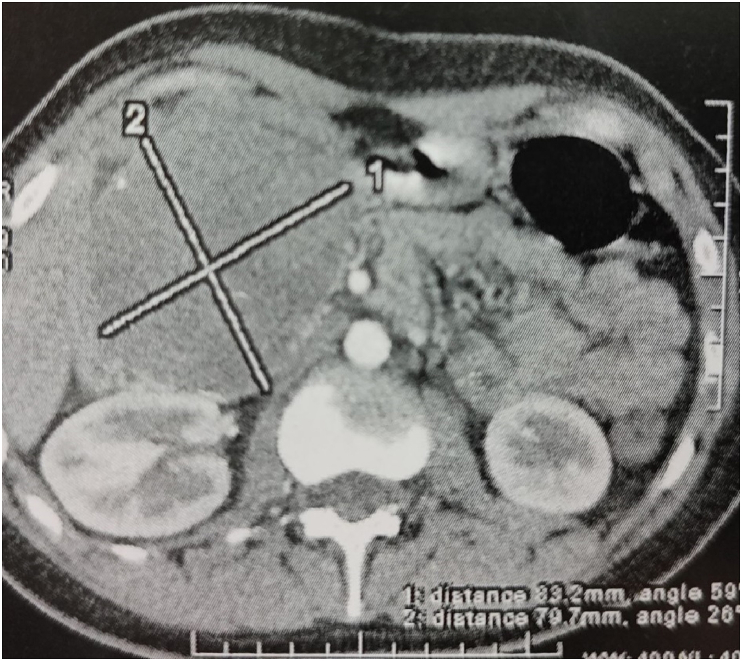
Abdominal CT demonstrates a well-defined mass in the head of the pancreas.

Abdominal Computed tomography (CT) scan showed the presence of a well-defined tumor arising from the pancreatic head measuring 9.1 × 8.1 × 8.5 cm, heterogeneous and with solid and necrosis components, with no pancreatic or ductal dilatation. No metastases were observed in adjacent organs (Fig. 1). Tumor markers and blood tests were normal.

**Fig. 2:**
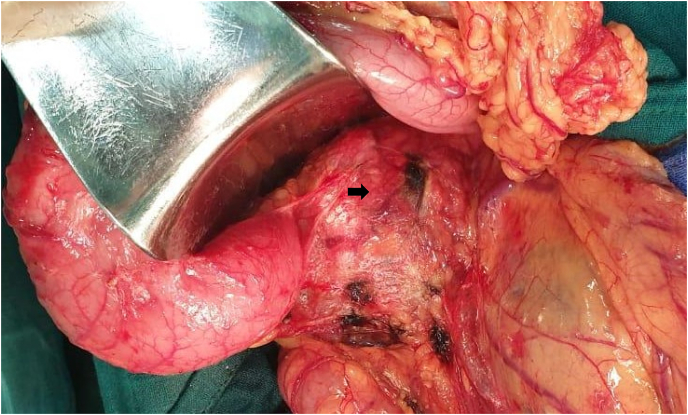
Intraoperative image showing the pancreatic the tumor (black arrow).

**Fig. 3:**
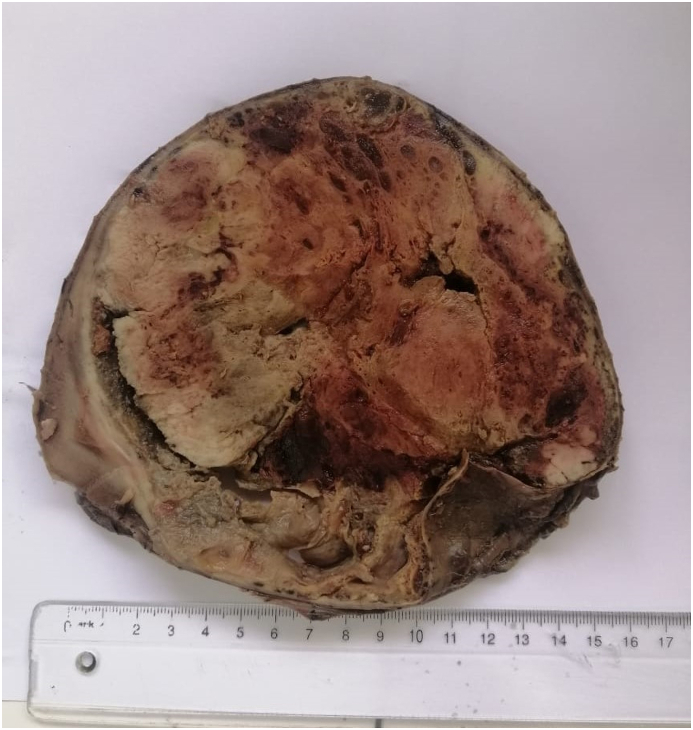
Gross picture of the cut section of tumor.

**Fig. 4:**
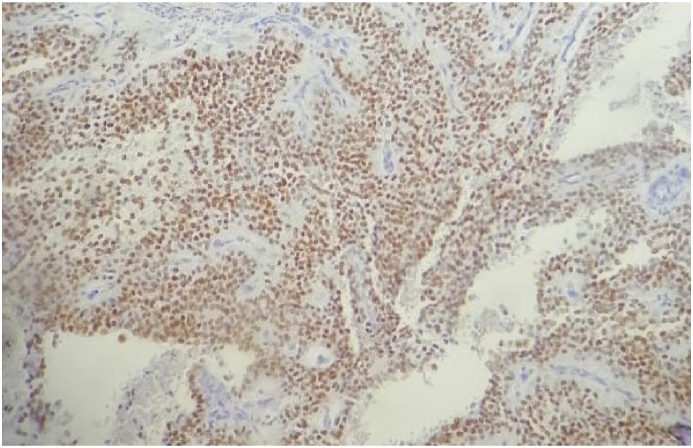
The tumor cells were positive for bêta-catenin.

During laparotomy under general anesthesia, a mass involving the head of the pancreas, measuring 9 cm, was noted (Fig. 2). The Liver and peritoneum were clear of metastases. The Patient underwent a Whipple procedure performed by senior general surgeon. On gross examination and sectioning, there was a well-circumscribed, encapsulated, solid, and cystic mass (9 × 9 × 9 cm) in the pancreatic head (Fig. 3). The Final histopathology report confirmed a solid pseudopapillary tumor, with 1.5 cm negative resection margins. Histopathology revealed a well-defined tumor with a fibrous pseudocapsule consisting of layers of polygonal cells with pseudopapillary formations. The immunohistochemistry was positive for vimentin, beta-catenin, CD10, with the focal expression of synaptophysin (Fig. 4).

Postoperatively, the patient had a pancreatic fistula and subcutaneous somatostatin analogs were administered at a dose of 100 μg three times a day for 7 days. The patient recovered from surgery and was discharged on postoperative day 20 in good condition. The patient was reviewed after 1 month, 3 months, and 6 months and continues to do well.

## 3 Discussion

SPN is classically described as an epithelial tumor with a macroscopic appearance of pseudo-papillary features and cystic microscopic appearance. The tumor has been identified with several synonyms, such as solid and papillary epithelial neoplasia, solid and cystic tumor, papillary cystic neoplasia, papillary cystic epithelial neoplasia, cystic papillary epithelial neoplasia tumor, and Franz tumor. In 1996, the World Health Organization (WHO) renamed this tumor SPT for the international histologic classification of tumors of the exocrine pancreas [5,6].

The common localization of solid pseudopapillary tumors is the tail of the pancreas, followed by the head and the body. More rarely it can stand as a multicentric tumor of the pancreas [2].

Its clinical presentation is nonspecific. The patients often are asymptomatic for a long time, until the tumor reaches a considerable size, because of symptoms associated with tumor compression of adjacent organs. Sometimes the patient may have mild abdominal pain, nausea, poor appetite, loss of weight, or palpable abdominal mass [7]. Jaundice or obstruction of the main pancreatic duct is uncommon, and clinical biochemistry is uncharacteristic and there are no useful tumor markers in plasma [2,8].

In the CT scan, the mass appears to be heterogeneous and hypodense. It is possible to search for calcifications. After injection, there is a peripheral capsular enhancement. The limits of the CT-scan remain the lack of tissue resolution [9]. Magnetic resonance imaging (MRI) is the method of choice for the evaluation of pancreatic pathologies due to its superior soft-tissue contrast [10]. The MRI typically shows a well-defined lesion with a mix of high and low signal intensity on T1-and T2-weighted images. Areas of high signal intensity on T1-weighted images and low or inhomogeneous signal intensity on T2-weighted images can help identify blood products and distinguish solid pseudopapillary tumors from other pancreatic tumors [11,12]. T2-weighted images show a thick fibrous capsule, which is seen as a discontinuous low signal boundary. Gadolinium-enhanced dynamic MRI shows early peripheral heterogeneous enhancement of the solid portion with progressive fill-in [12].

The problem of the differential diagnosis is above all raised with neuroendocrine tumors of the pancreas and mucinous cystadenocarcinomas where the prognosis is less favorable [13].

Histologically, macroscopically, the lesion is separated from the healthy parenchyma by a thin fibrous capsule. Microscopically, the cystic zones are surrounded by solid tumoral tissue presenting characteristic pseudopapillary structures, with the progression of the tumor necrotic and hemorrhagic zones developing [9].

The tumors show immunohistochemical stains for vimentin, alpha 1- antitrypsin, neuron-specific enolase, CD56, and CD10 [14]. Positivity staining for chromogranin is never seen.

The genetic pathway for NPS formation is well known, and NPS is characterized by the presence of mutations in the bêta-catenin activating gene that interfere with the phosphorylation of the protein product [15].

Surgical resection is the treatment of choice for SPN and organ preservation is advocated if feasible. According to the location of the tumor, distal pancreatectomy with or without splenectomy, pylorus-preserving pancreatoduodenectomy, Whipple operation can be performed [16]. Enucleation can be done for small SPN distant from the pancreatic duct, but it is associated with a high risk of pancreatic fistula [17]. Lymphadenectomy is not recommended since lymph node metastases are extremely rare [18]. In about 10%–15% of cases, SPNs are metastatic to the liver or peritoneum, Metastasectomy of the liver is advocated at the time of primary resection or even for the recurrences when feasible [17,19].

Long-term survival and cure for SPN are achieved through surgical resection, The overall five-year survival rate of patients with SPN is about 95% [2]. Malignant SPN, designated as a solid pseudopapillary carcinoma, occurs in 15% of adult patients. SPT was classified according to the WHO as either solid pseudopapillary neoplasms with borderline malignancy or solid pseudopapillary carcinoma SPC [6].

Criteria that distinguish potentially malignant tumors and which are classified as SPC carcinoma are angioinvasion, perineural invasion, and deep invasion of the surrounding pancreatic parenchyma. Other histological features, such as extensive necrosis, nuclear atypia, high mitotic rate, Ki-67 proliferative index, and sarcomatoid areas may be associated with aggressive behavior [14,16].

## 4 Conclusion

SPT of the pancreas is a rare tumor with low malignant potential, but the incidence is increasing. Large tumors of the pancreas among young women with solid and cystic areas should be highly suspected as SPT. The prognosis is favorable even in the presence of distant metastasis. Although surgical resection with clear margins seems to be the best surgical strategy in the management of SPT. The postoperative follow-up strategy should be personalized based on the presence of risk factors to diagnose a local recurrence or distant metastasis.

## Provenance and peer review

Not commissioned, externally peer-reviewed.

## Ethical approval

I declare on my honor that the ethical approval has been exempted by my establishment.

## Sources of funding

None.

## Author contribution

Driss Erguibi: written the paper and operating surgeon. Yassine El Berni: written the paper. Aziz Moufakkir: written the paper. Rachid Boufettal: study concept. Rifki Jai Saad: study concept. Farid Chehab: correction of the paper.

## Consent

Written informed consent for publication of their clinical details and/or clinical images was obtained from the patient. A copy of the written consent is available for review by the Editor-in-Chief of this journal on request.

## Research registration

None.

## Guarantor

Yassine El Berni.

## Declaration of competing interest

The authors declare having no conflicts of interest for this article.

## References

[1] Papavramidis T., Papavramidis S. (2005). Solid pseudopapillary tumors of thepancreas: review of 718 patients reported in English literature. J. Am. Coll. Surg..

[2] de Lagausie P., Sarnacki S., Orbach D., Petit P., Joubert C. (2012). Tumeur cystopapillaire du pancréas (tumeur de Frantz) chez l’adolescent et l’adulte jeune. Bull. Cancer.

[3] Agha R.A., Franchi T., Sohrabi C., Mathew G., For the SCARE Group (2020). The SCARE 2020 Guideline: updating consensus surgical CAse REport (SCARE) Guidelines. Int. J. Surg..

[4] Vassos Nikolaos, Agaimy Abbas, Klein Peter, Hohenberger Werner, Croner Roland S. (2013). Solid pseudo-papillary neoplasm (SPN) of the pancreas: case series and literature review on an enigmatic entity. Int. J. Clin. Exp. Pathol..

[5] Frantz V. Tumors of the pancreas. In: Atlas of Tumor Pathology, Section 7, Fascicles 27 and 28. Washington, DC; Armed Forces Institute of Pathology.

[6] Kloppel G., Solcia E., Longnecker D.S. (1996). Histological Typing of Tumors of the Exocrine Pancreas. WHO International Histological Classification of Tumors.

[7] Casanova M., Collini P., Ferrari A. (2003). Solid pseudo-papillary tumor of the pancreas (Frantz tumor) in children. Med. Pediatr. Oncol..

[8] Reddy S., Cameron J.L., Scudiere J., Hruban R.H., Fishman E.K., Ahuja N., Pawlik T.M., Edil B.H., Schulick R.D., Wolfgang C.L. (2009). Surgical management of solid- pseudo-papillary neoplasms of the pancreas: a large single-institutional series. J. Am. Coll. Surg..

[9] Romics L., Oláh A., Belágyi T., Hajdú N., Gyurus P., RuszinkóV (2010). Solid pseudopapillary neoplasm of the pancreas-proposedalgorithms for diagnosis and surgical treatment. Langenbeck's Arch. Surg..

[10] Franz D., Esposito I., Kapp A.-C., Gaa J., Rummeny E.J. (2014). Magnetic resonance imaging of less common pancreatic malignancies and pancreatic tumors with malignant potential. European Journal of Radiology Open.

[11] Coleman K.M., Doherty M.C., Bigler S.A. (2003). Solidpseudopapillary tumor of the pancreas. Radiographics.

[12] Choi Jin-Young, Kim Myeong-Jin, Hee Joo, Kim (2006). Solid pseudopapillary tumor of the pancreas: typical and atypical manifestations. Am. J. Roentgenol..

[13] Kalb B., Sarmiento J.M., Kooby D.A., Adsay N.V., Martin D.R. (2009). MR imaging of cystic lesions of the pancreas. Radiographics.

[14] Tang L.H., Aydin H., Brennan M.F., Klimstra D.S. (2005). Clinically aggressive solid pseudo-papillary tumors of the pancreas. A report of two cases with components of undifferentiated carcinoma and a comparative clinicopathologic analysis of 34 conventional cases. Am. J. Surg. Pathol..

[15] Abraham S.C., Klimstra D.S., Wilentz R.E. (2002). Solid-pseudopapillary tumors of the pancreas are genetically distinct from pancreatic ductal adenocarcinomas and almost always harbor bêta-catenin mutations. Am. J. Pathol..

[16] Yagcı A., Yakan S., Coskun A. (2013). Diagnosis and treatment of solid pseudopapillary tumor of the pancreas: experience of one single institution from Turkey. World J. Surg. Oncol..

[17] Gandhi Lanke, Faisal S Ali, Jeffrey H Lee. Clinical update on the management of pseudopapillary tumor of pancreas. World J. Gastrointest. Endosc. 10(9), 145–155. 10.4253/wjge.v10.i9.145 PMC6162250 30283597

[18] Klotz D Da Ines, Petitcolin V., Lannareix V., Essamet W., Garcier J.-M. (2013). Solid pseudopapillary neoplasm of the pancreas. Diagnostic and Interventional Imaging.

[19] Rosa S.L., Bongiovanni M. (2020). Pancreatic solid pseudopapillary neoplasm: key pathologic and genetic features. Arch. Pathol. Lab Med..

